# Cervical Cancer Cells-Derived Extracellular Vesicles Containing microRNA-146a-5p Affect Actin Dynamics to Promote Cervical Cancer Metastasis by Activating the Hippo-YAP Signaling Pathway *via* WWC2

**DOI:** 10.1155/2022/4499876

**Published:** 2022-06-28

**Authors:** Weiwei Wang, Lipei Wu, Jiale Tian, Wenhui Yan, Chunrun Qi, Wuchao Liu, Shihai Xuan, Anquan Shang

**Affiliations:** ^1^Department of Laboratory Medicine, Shanghai Tongji Hospital, School of Medicine, Tongji University, Shanghai 200065, China; ^2^Department of Pathology, Tinghu People's Hospital, Yancheng 224005, China; ^3^Department of Laboratory Medicine, Dongtai People's Hospital, Yancheng 224200, China; ^4^Department of Neurorehabilitation, Yangzhi Rehabilitation Hospital (Shanghai Sunshine Rehabilitation Center), Tongji University School of Medicine, Shanghai 201619, China

## Abstract

Application of extracellular vesicles (EVs) for cancer treatment has been well-documented. We probed into the potential role of cervical cancer cells-secreted EVs by transferring miR-146a-5p in cervical cancer. After characterization of miR-146a-5p expression in clinical cervical cancer tissue samples, gain- and loss-of-function experiments were implemented to test the effect of miR-146a-5p on the invasion, epithelial-mesenchymal transition (EMT), and anoikis in cervical cancer cells. EVs were isolated from high-metastatic cervical cancer cells, after which their effects on the malignant behaviors of low-metastatic cervical cancer cells were assessed in a co-culture system. Luciferase assay was implemented to validate the putative binding relationship between miR-146a-5p and WWC2, followed by further investigation of downstream pathway (Hippo-YAP). Finally, nude mouse lung metastasis model was developed for *in vivo* validation. miR-146a-5p was elevated in cervical cancer tissues and high miR-146a-5p expression promoted the metastatic potential of cervical cancer cells through enhancing their invasiveness and anoikis resistance, and inducing EMT. Furthermore, miR-146a-5p carried by EVs secreted by highly metastatic cervical cancer cells could promote the metastasis of low-metastatic cervical cancer cells. Mechanistically, miR-146a-5p targeted WWC2 to activate YAP, by which it inhibited the phosphorylation of cofilin, and promoted the process of cofilin-mediated depolymerization of F-actin to G-actin. *In vivo* data demonstrated that EVs-carried miR-146a-5p promoted tumor metastasis through the WWC2/YAP axis. Cancer-derived EVs delivered pro-metastatic miR-146a-5p to regulate the actin dynamics in cervical cancer, thereby leading to cancer metastasis. This experiment highlighted an appealing therapeutic modality for cervical cancer.

## 1. Introduction

Cervical cancer poses a main gynecological problem in both developing and underdeveloped countries [1]. Recent advancements in imaging diagnosis, management, and treatment contribute to the global improvement of 5-year net survival of cervical cancer patients [2]. Metastasis remains as the major reason for the treatment failures of most patients with cervical cancer [3]. Though various pathologies associated with cervical cancer progression have been demonstrated, further investigation involving tumor metastasis is still necessary [4, 5].

Extracellular vesicles (EVs) have attracted much attention from oncologists owing to their promising potential as prognostic indicators [6]. It is known that EVs containing messengers such as microRNAs (miRs) bear great responsibility in modulating the tumor development and metastasis [7]. Importance of miRs in tumor oncogenesis and metastasis has been well-characterized [8]. Specifically, elevated miR-146a-5p is witnessed in the EVs derived from inflammatory microglia [9]. Furthermore, miR-146a can enhance the viability of cervical cancer cells through regulating IRAK1 and TRAF6 [10]. More recently, upregulation of miR-146a-5p is identified in the plasma EVs of patients with cervical cancer, and it can be a promising novel bio-marker for the diagnosis of cervical cancer [11]. Based on the aforementioned findings, whether EVs containing miR-146a-5p involves in cervical cancer is yet to be investigated.

All WW-and-C2-domain-containing (WWC) proteins in the WWC protein family can suppress the transcriptional activity of YAP to inhibit cell proliferation and organ growth [12]. It is reported that WWC2, belonging to the WWC protein family, exerts tumor-suppressing function in lung adenocarcinoma through negatively regulating the Hippo signaling pathway [13]. Emerging evidence shows the involvement of WWC2 in cancer metastasis. For instance, WWC2 can reverse epithelial-mesenchymal transition (EMT) and block stemness maintenance of pancreatic cancer stem cells through inhibiting the Hippo signaling pathway [14]. The Hippo-YAP signaling pathway exerts a great function in regulating organ volume, stem cells, tissue regeneration, and tumorigenesis [15]. It is reported that the Hippo/YAP signaling pathway can regulate cervical cancer progression through interacting with EGFR signaling and HPV oncoproteins [16]. YAP functions as a promoter of tumor metastasis through regulating the actin dynamics [17].

In view of these findings, this study is intended to explore the regulatory mechanism how the cervical cancer cell-secreted EVs carrying miR-146a-5p contributes to cervical cancer metastasis.

## 2. Materials and Methods

### 2.1. Ethics Statement

All individuals involved in this study signed informed consent. The study was ratified by Ethics Committee of Shanghai Tongji Hospital, School of Medicine, Tongji University (No. 2021-KYSB-161) and in line with the ethical standards of the Declaration of Helsinki. Animal experiment was ratified by the Animal Ethics Committee of Shanghai Tongji Hospital, School of Medicine, Tongji University (No. 2021-DWSB-079).

### 2.2. Bioinformatics Analysis

The cervical cancer-related miRNA expression microarray GSE86100 was obtained through retrieval from the Gene Expression Omnibus database, which contained a total of 12 samples (6 normal samples and 6 cervical cancer samples). The R “limma” software package was run for differential analysis with |logFC| > 1 and *p* < 0.05 used as thresholds to screen the significantly differentially expressed miRNAs in the microarray. In addition, miRNA expression microarray GSE30656 including 10 normal samples and 19 cervical cancer samples were obtained for miRNA expression verification. The ENCORI, miRDB, miRWalk, and TargetScan databases were searched to predict target genes of miR-146a-5p utilizing |log2FC| > 1.5 and *p* value <0.01 as the screening criteria. The cervical cancer-related genes were obtained through the GEPIA2 website, which were intersected with the target genes to predict downstream regulatory genes of miR-146a-5p in cervical cancer.

### 2.3. Clinical Sample Collection

Cervical cancer tissues were collected from 30 patients diagnosed with cervical cancer with an average age of 52.7 ± 6.25 from the Shanghai Tongji Hospital, School of Medicine, Tongji University. In accordance with the diagnostic criteria of the International Federation of Gynecology and Obstetrics systems (2014), all patients were examined by two gynecological oncologists with senior seniority and above through combined gynecological ultrasound, pelvic magnetic resonance imaging, computed tomography, and other examinations. Inclusion criteria were: (1) Histologically or cytologically confirmed cervical cancer; (2) Patients with primary cervical cancer were treated at the first time; (3). Pathological types: squamous cell carcinoma, adenocarcinoma, and adenosquamous cell carcinoma; (4) Patients showed no other tumors or treatment history of other tumors. Exclusion criteria were: (1) Neuroendocrine cervical tumor or other rare histology (such as clear cell carcinoma) through pathological diagnosis; (2) Patients were not newly diagnosed cancer patients; (3) Patients had history of tumor treatment; (4) Patients undergoing drug intervention. Normal cervical tissues were collected from 30 patients (mean age of 49.67 ± 5.65) who were diagnosed with uterine fibroids without HPV infection or cervical lesions. The tissue samples were stored at −80°C.

### 2.4. Cell Culture and Lentivirus Transduction

Human embryonic kidney cells (HEK293T cells), cervical cancer cell lines (HeLa, CaSki, SiHa, and C33A), and normal cervical epithelial cell lines (End1/E6E7 and HcerEpic) were all procured from Mingzhou Biotechnology (Ningbo, China). The cells were cultured in Dulbecco's modified Eagle's medium (DMEM) (Gibco, Grand Island, NY) replenishing 10% fetal bovine serum (Gibco), 100 U/mL penicillin, and 100 *μ*g/mL streptomycin (Gibco) at 37°C with 5% CO_2_. miR-146a-5p mimic, miR-146a-5p inhibitor (this type of inhibitor is chemically modified specifically for the specific target miR-146a-5p in cells, which can specifically target and knock down the expression of a single miRNA molecule), and negative controls (mimic-NC and inhibitor-NC) were all procured from GenePharma Co., Ltd. (Shanghai, China).

The cells were transduced with Vector, WWC2, short hairpin RNA (sh) NC, shWWC2-1, shWWC2-2, shYAP-1, shYAP-2, and shWWC2 + shYAP, respectively. Logarithmically growing cells were detached with trypsin, prepared into cell suspension (5 × 10^4^) cells/mL and seeded in a 6-well plate with 2 mL per well, followed by culture overnight at 37°C. After 48 h of transduction, the GFP expression efficiency was checked by a fluorescence microscope.

The stable-transduced cell line was constructed. At 72 h after the virus transduction, the medium was renewed with a complete medium appended to 2 *μ*g/mL puromycin. The shRNA sequence (Supplementary Table 1) was designed by Life Technologies and synthesized by GenePharma.

### 2.5. Extraction and Identification of EVs

The equal number of well-grown cervical cancer cells CaSki were collected, plated in a 10 cm plate, and cultured overnight in DMEM containing 10% EV-free serum (overnight centrifugation was performed at 12,000 × *g* to remove EVs). Under 60–70% confluence, the supernatant was harvested. The clinical serum samples and cell culture supernatant were centrifuged at 2000*g* and 4°C for 20 minutes to remove cell debris, and the resulting supernatant was filtered utilizing a 0.22 *μ*m filter (Merck Millipore, Billerica, MA). EVs were gained by ultracentrifugation as previously described [18]. Ultracentrifugation was processed utilizing Optima Max-XP (Beckman Coulter, Miami, FL).

The EVs were observed under a Philips CM120 BioTwin transmission electron microscope (FEI company). The EV particles were used for quantitative identification. The antibodies (Abcam, Cambridge, UK) for Western blot assay included TSG101 (ab125011, 1 : 1000), CD63 (ab134045, 1 : 1000), CD81 (ab109201, 1 : 5000), and Calnexin (ab22595). Diameter of EVs was determined employing dynamic light scattering utilizing Zetasizer Nano-ZS90 instrument of Malvern Company (Malvern, UK).

### 2.6. Uptake of EVs

The extracted CaSki EVs were labeled by referring to the PHK67 labeling kit (KH67GL, Sigma-Aldrich, St Louis, MO). C33A cells were cultured overnight in the Petri dish with a special cell climbing piece and then incubated with 10 *μ*g PHK67-labeled CaSki-derived EVs for 24 h. Following soaked in 4% paraformaldehyde for 30 minutes, the slices were subjected to permeabilization with 2% Triton X-100 for 15 minutes and blockage with 2% bovine serum albumin (BSA) for 45 minutes. After that, the slides were stained with 4′,6-diamidino-2-phenylindole (DAPI) (2 *μ*g/mL) and observed under an inverted fluorescence microscope.

### 2.7. Co-Culture of Cy3-Labeled CaSki Cells and C33A Cells

CaSki cells were transduced with Cy3-labeled miR-146a-5p (miR-146a-5p-Cy3, GenePharma) utilizing the Lipofectamine 2000 transfection reagent (11668019, Invitrogen Inc. Carlsbad, CA, USA) for identifying the transfer of EVs-derived miR-146a-5p. The CaSki cells expressing Cy3-miR-146-5p were seeded into a 6-well plate and co-cultured with C33A cells in a Transwell chamber (3412, Corning Glass Works, Corning, NY) for 2 to 4 days. Finally, the cells were stained with DAPI (C1002, Beyotime, Shanghai, China) for 5 minutes and observed using a confocal microscope.

### 2.8. RT-qPCR

TRIzol (16096020, Invitrogen) was employed to extract total RNA from tissues and cells. For miRNA, PolyA tailing detection kits (B532451, Sangon Biotech Co., Ltd. Shanghai, China) containing universal PCR primer R and U6 universal PCR primer R were adopted to obtain cDNA of miRNA with PolyA tail. Using SYBR Premix Ex Taq™ II kits (DRR081, Takara, Kyoto, Japan), RT-qPCR was run in a real-time fluorescent quantitative PCR instrument (ABI 7500, ABI, Foster City, CA). U6 was served as a normalizer for miRNA in tissues while syn-cel-miR-39 (miRB0000010-3-1, RiboBio, Guangzhou, China) as an internal control in EVs.

For mRNA detection, cDNA was obtained employing reverse transcription kits (RR047A, TaKaRa, Tokyo, Japan). On the basis of TaqMan Gene Expression Assays protocol (Applied Biosystems, Foster City, CA), PCR was conducted. GAPDH was used as a normalizer. Primer sequences are described in Supplementary Table 2. The relative expression was quantified utilizing the 2^−ΔΔCt^ method.

### 2.9. Western Blot Assay

For immunoblotting of targeted proteins, the following antibodies were used: YAP [1 : 1000, ^#^14074, Cell Signaling Technologies (CST), Beverly, MA], WWC2 (1.0 *μ*g/mL, PA5-71284, Invitrogen), E-cadherin (1.0 *μ*g/mL, ab231303, Abcam), N-cadherin (1.0 µg/mL, ab18203, Abcam), Vimentin (1 : 1000, ab137321, Abcam), LATS1 (1 : 1000, ^#^3477, CST), phosphorylated (p)-LATS1 (1 : 1000, ^#^8654, CST), p-YAP (1 : 1000, ^#^4911, CST), cofilin (1.0 *μ*g/mL, ab42824, Abcam), p-cofilin (1 : 1000, ab12866, Abcam), F-actin (1 : 1000, ab205, Abcam), G-actin (1 : 1000, ab123034, Abcam), and GAPDH (1 : 5000, ab8245, Abcam) as well as horseradish peroxidase labeled goat anti-rabbit IgG (ab6721, Abcam) or goat anti-mouse IgG (ab6789, Abcam). ImageJ software was utilized for protein quantitative analysis.

### 2.10. Immunofluorescence and Cytoskeleton Staining

For immunofluorescence, the cells were incubated with anti-YAP (14074, CST) antibody overnight at 4°C, with secondary antibody (Proteintech) for 1 h, and stained with DAPI (C1002, Beyotime) solution for 5 minutes. For cytoskeleton staining, the cells were incubated with Phalloidin-FITC (P5282, Sigma-Aldrich, 50 *μ*g/mL) for 30–90 minutes (dark condition). Under the FV-1000/ES confocal microscope, five different fields of view were selected for observation.

### 2.11. Dual-Luciferase Reporter Gene Assay

The wild-type (wt) plasmid containing WWC2 3′untranslated region (UTR) full-length sequence and mutant (mut) plasmid were constructed and designated as PmirGLO-WWC2-wt and PmirGLO-WWC2-mut, respectively. The dual luciferase reporter plasmid and the corresponding plasmid were co-transfected into 293T cells. After 48 h of transfection, the luciferase activity was assayed employing Dual-Luciferase® Reporter Assay System (Dual-Luciferase® Reporter Assay System, E1910, Promega, Madison, WI).

### 2.12. Transwell Assay

Transwell chambers (pore size of 8 mm; Corning Incorporated, Corning, NY, USA) in 24-well plates were adopted for invasion and migration experimentations. A total of 100 *μ*L of Matrigel was spread in each chamber and incubated at 37°C for 2 h for invasion detection. Matrigel was not included in cell migration experiment. The cells were treated with the equal number of EVs in different groups, detached, and resuspended in serum-free DMEM (Gibco) to a density of 3 × 10^5^ cells/mL [19]. The invaded or migrative cells were counted and photographed under an Olympus IX 71 microscope (Olympus Optical Co., Ltd, Tokyo, Japan).

### 2.13. Immunohistochemistry

After preparation of tissue sections, deparaffinization with xylene and blockage of endogenous peroxidase activity utilizing 3% hydrogen peroxide were implemented. Then, the sections were blocked in 10% normal goat serum for 15 minutes. The antibody YAP (1 : 200; ^#^14074, CST) was added for incubation overnight at 4°C. The next day, the secondary antibody was added to the sections for 1-h of incubation. The immunoreactivity was quantified utilizing the diaminobenzidine tetrahydrochloride kit (Invitrogen), followed by a microscopic observation.

### 2.14. TUNEL Assay for Cell Apoptosis

Cell apoptosis was detected with TUNEL kits (C1089, Beyotime) and observed under a FV-1000/ES confocal microscope [20].

### 2.15. Cell-Matrix Adhesion Test

Firstly, a 96-well plate was coated with 10 mg/mL FN1/fibronectin (10838039001, Sigma-Aldrich) at 4°C, and then 1% BSA (A7030, Sigma-Aldrich) was added to stop the reaction. A total of 5 × 10^4^ cervical cancer cells were incubated at 37°C for 10 minutes, and fixed with 4% paraformaldehyde for 30 minutes. Then, the cells were stained with 0.5% crystal violet (G1062, Solarbio) for 10 minutes and treated with 30% glacial acetic acid (A116172, Aladdin Equipment Company, Inc, Shanghai, China) for 15 minutes. Subsequently, the cells were photographed under the microscope (Olympus IX 71, Olympus).

### 2.16. Nude Mouse Lung Metastasis Model and *In Vivo* Imaging

The healthy female BALB/c nude mice aged 4–6 weeks (National Rodent Laboratory Animal Resource, Shanghai Branch, PRC) were separately caged in a specific-pathogen-free animal laboratory for 1 week. The C33A cells stably transduced with Vector, WWC2, shNC, or shYAP were trypsinized and dispersed into single cell suspension. A total of 5 × 10^5^ luciferase-labeled stable-transduced cells were injected into nude mice through the tail vein. At the same time, 20 *μ*g EVs or PBS was injected through the tail vein every three days. Mice were intraperitoneally injected with 200 *μ*L of luciferase substrate D-luciferin (15 mg/mL; Gold Biotechnology, St. Louis, MO), and 15 minutes later, imaged on an *in vivo* animal imager (UVP iBox® Scientia™, Germany). The survival curve of nude mice was plotted using GraphPad software.

### 2.17. Hematoxylin and Eosin (H&E) Staining

According to H&E staining kits (C0105, Beyotime), the tissue samples were dewaxed in xylene, hydrated with gradient ethanol, stained with hematoxylin (5–10 minutes), and with eosin (30 s to 2 minutes). After that, the samples were observed under a microscope (Olympus IX 71, Olympus).

### 2.18. Statistical Analysis

GraphPad Prism software was processed for data analysis, and the *p* value was determined by a two-tailed *t*-test on independent samples. Measurement data were expressed as mean ± standard deviation. Comparison between the two groups was performed by independent sample *t*-test, and comparison among multiple groups was assayed by one-way analysis of variance. The Kaplan–Meier method was utilized to calculate the survival rate of nude mice, and the Log-rank test was applied for univariate analysis. *p* < 0.05 indicated statistical significance.

## 3. Results

### 3.1. miR-146a-5p Was Highly Expressed in Cervical Cancer and Enhanced Malignancy of Cervical Cancer Cells

**Figure 1:**
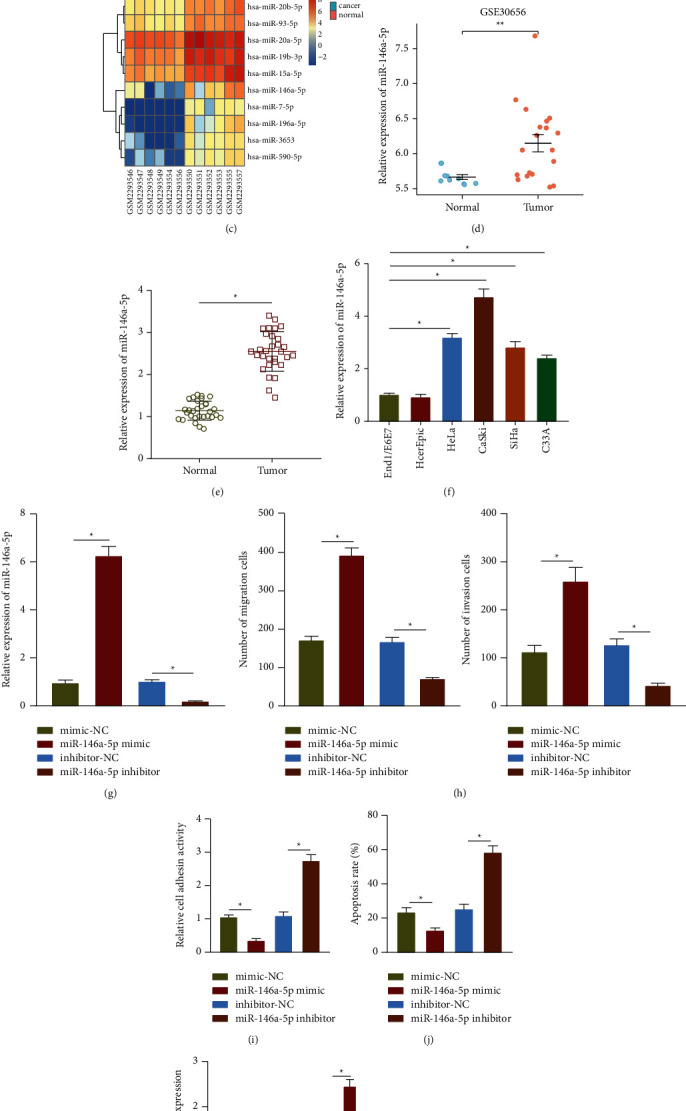
miR-146a-5p shows an upregulation in cervical cancer and exerts promotive effects on metastasis of cervical cancer cells. (a) The heat map of differentially expressed miRNAs in microarray GSE86100. Each row represents a differentially expressed miRNA, and each column represents a sample. (b) The volcano map of differentially expressed miRNAs in microarray GSE86100, where the red dots indicate upregulated miRNAs and the green dots indicate downregulated miRNAs. (c) The heat map of top 10 upregulated miRNAs in microarray GSE86100. (d) The expression of miR-146-5p in microarray GSE30656. (e) RT-qPCR determination of miR-146a-5p expression in 30 cases of human cervical cancer tissues and 30 cases of normal cervical tissues. (f) RT-qPCR determination of the expression of miR-146a-5p in cervical cancer cell lines (HeLa, CaSki, SiHa, and C33A) and normal cervical epithelial cell lines (End1/E6E7 and HcerEpic). (g) RT-qPCR determination of the expression of miR-146a-5p in C33A cells after transfection. (h) Transwell assay of the invasion and migration of C33A cells after miR-146a-5p mimic or inhibitor transfection. (i) The adhesion of C33A cells to the extracellular matrix after miR-146a-5p mimic or inhibitor transfection. (j) The apoptosis of C33A cells after miR-146a-5p mimic or inhibitor transfection assessed by TUNEL staining. (k) Western blot assay of E-cadherin, N-cadherin, and Vimentin protein expression in C33A cells after miR-146a-5p mimic or inhibitor transfection. The data in the Figure were measurement data and expressed as mean ± standard deviation. The data between two groups were analyzed by unpaired *t*-test, and the comparison among multiple groups was performed by a one-way analysis of variance (Dunnett's post hoc test). ^*∗*^*p* < 0.05, ^*∗∗*^*p* < 0.01. Cell experiments were repeated 3 times independently.

In order to clarify the specific molecular mechanism of miRNAs affecting cervical cancer, we first analyzed the differential miRNA expression in cervical cancer microarray GSE86100. The results showed 92 upregulated miRNAs and 75 downregulated miRNAs in cervical cancer (Figures 1(a) and 1(b)). The expression heat map (Figure 1(c)) shows the top 10 upregulated miRNAs in cervical cancer samples, including hsa-miR-7-5p, hsa-miR-146a-5p, hsa-miR-20b-5p, hsa-miR-19b-3p, hsa-miR-196a-5p, hsa-miR-93-5p, hsa-miR-15a-5p, hsa-miR-3653, hsa-miR-590-5p, and hsa-miR-20a-5p. As recently documented, miR-146a-5p is elevated in cervical cancer and promotes cell migration and invasion, but the specific mechanism remains unclear [21]. Following analysis of microarray GSE30656 also found elevated miR-146a-5p in cervical cancer tissues (Figure 1(d)).

Subsequent RT-qPCR identified similar elevation of miR-146a-5p expression in cervical cancer tissues (Figure 1(e)). Meanwhile, its elevation was also witnessed in HeLa, CaSki, SiHa, and C33A cells than in End1/E6E7 and HcerEpic cells. Among all cancer cells, miR-146a-5p expressed at the highest level in high-metastatic CaSki cells and at the lowest level in low-metastatic C33A cells (Figure 1(f)).

We then probed into the effect of miR-146a-5p on cervical cancer metastasis. RT-qPCR results showed increased expression of miR-146a-5p in the cells transfected with miR-146a-5p mimic but opposite finding was witnessed upon miR-146a-5p inhibitor transfection (Figure 1(g)). Additionally, mimic of miR-146a-5p promoted cell migration and invasion, while loss-of-function of miR-146a-5p caused contrary trends (Figure 1(h), Supplementary Figure 1A). It has been shown that cancer cells are separated from the extracellular matrix at the primary site or neighboring cells during metastasis, and circulated to a distant place to escape apoptosis during transport (i.e., anti-anoikis) [21]. Therefore, the cell-matrix adhesion experiment was conducted to assess the adhesion of cells to the extracellular matrix. We found that mimic of miR-146a-5p could inhibit cell adhesion to the extracellular matrix, while loss-of-function of miR-146a-5p promoted their adhesion to the extracellular matrix (Figure 1(i), Supplementary Figure 1B). At the same time, we assessed cell apoptosis after anoikis treatment (to mimic the microenvironment of extracellular matrix loss). TUNEL assay results depicted that the number of anoikis of cells overexpressing miR-146a-5p was decreased, but it was increased following miR-146a-5p inhibition (Figure 1(j), Supplementary Figure 1E). Next, the EMT marker protein expression was characterized by Western blot assay, which clarified that overexpression of miR-146a-5p diminished E-cadherin expression and promoted the expression of N-cadherin and Vimentin; while loss-of-function of miR-146a-5p brought about opposing tendency (Figure 1(k), Supplementary Figure 1F). Additionally, we overexpressed miR-146a-5p in HcerEpic cells, and determined the effect of miR-146a-5p on the migration and invasion of HcerEpic. The obtained results were similar to those of C33A cells, but gain-of-function of miR-146a-5p had a small effect on cell migration and invasion (Supplementary Figures 2A–2D). Western blot assay results indicated that miR-146a-5p mimic in HcerEpic cells diminished E-cadherin expression while promoting that of N-cadherin and Vimentin (Supplementary Figure 2E).

In order to elucidate whether other miRNAs have an impact on cervical cancer metastasis, we initially performed RT-qPCR to analyze the expression of the top two upregulated miRNAs hsa-miR-7-5p and hsa-miR-20b-5p in clinical cervical cancer tissues. The results displayed an elevation in the expression of the two miRNAs in the cervical cancer tissues (Supplementary Figure 3A). Furthermore, RT-qPCR results confirmed higher expression of miR-7-5p and miR-20b-5p in cervical cancer cell lines. Among all cancer cells, miR-7-5p and miR-20b-5p expressed at the highest level in high-metastatic CaSki cells and at the lowest level in low-metastatic C33A cells (Supplementary Figure 3B). Besides, we silenced or overexpressed miR-7-5p and miR-20b-5p in C33A cells, followed by determination of silencing and overexpression efficiency utilizing RT-qPCR (Supplementary Figure 3C). Moreover, the results of Transwell assay unveiled that overexpression or silencing of miR-7-5p/miR-20b-5p had no effect on cell migration and invasion (Supplementary Figures 3D and 3E). Cell-matrix adhesion test data indicated that the overexpression or silencing of miR-7-5p/miR-20b-5p could not promote cell adhesion to the extracellular matrix (Supplementary Figure 3F). At the same time, we performed anoikis treatment on the cells (to mimic the microenvironment of the absence of extracellular matrix), and then used TUNEL assay to detect cell apoptosis. The results suggested that neither hsa-miR-7-5p nor hsa-miR-20b-5p overexpression or inhibition showed effect on the number of anoikis (Supplementary Figure 3G).

Conclusively, miR-146a-5p was elevated in cervical cancer, and its overexpression could promote malignant and anti-anoikis abilities of cervical cancer cells while inhibiting cell adhesion to the extracellular matrix.

### 3.2. Cervical Cancer Cells-Derived EVs Carried miR-146a-5p to Promote the Metastasis of Cervical Cancer

**Figure 2:**
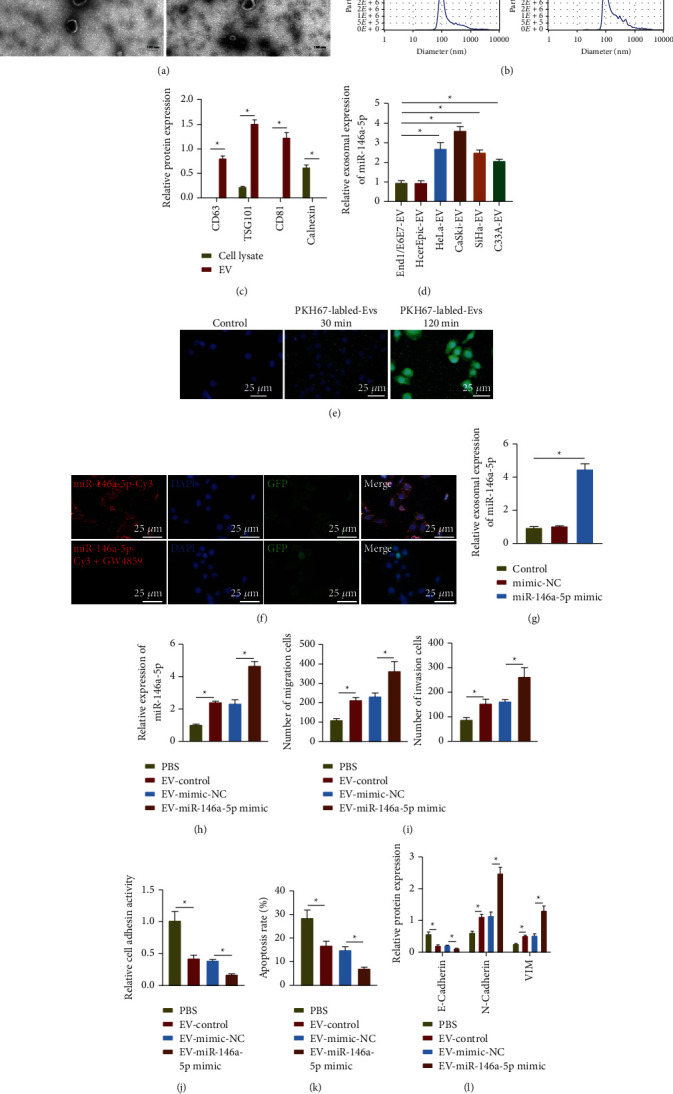
EVs secreted by high-metastatic cervical cancer cells carrying miR-146a-5p can promote the malignancy of low-metastatic cervical cancer cells. (a) Transmission electron microscopy for identification of the EVs isolated from cervical cancer cell line (CaSki) and normal cervical epithelial cell line (HcerEpic). (b) Dynamic light scattering detection of the diameter of EVs isolated from cervical cancer cell line (CaSki) and normal cervical epithelial cell line (HcerEpic). (c) Western blot assay of the expression of EV surface markers (CD81, CD63, and TSG101) and endoplasmic reticulum marker protein Calnexin. (d) RT-qPCR determination of the expression of miR-146a-5p in EVs secreted by cervical cancer cell lines (HeLa, CaSki, SiHa, and C33A) and normal cervical epithelial cells (End1/E6E7 and HcerEpic). (e) The uptake of CaSki cells-secreted EVs by C33A cells observed under an inverted fluorescence microscope, scale bar: 25 *μ*m. (f) C33A cells transfected with pCDNA3.1-GFP after co-culture with CaSki cells transfected with miR-146a-5p-Cy3 observed using an inverted fluorescence microscope, scale bar: 25 *μ*m. (g) RT-qPCR determination of the expression of miR-146a-5p in EVs secreted by different groups of CaSki cells. (h) RT-qPCR determination of the expression of miR-146a-5p in C33A cells after treatment with EV-miR-146a-5p-mimic. (i) Transwell assay of invasion and migration of C33A cells after treatment with the equal number of EVs from miR-146a-5p-mimic-transfected CaSki cells. (j) The adhesion of C33A cells to the extracellular matrix after treatment with EV-miR-146a-5p-mimic. (k) TUNEL staining of the apoptosis of C33A cells after treatment with EV-miR-146a-5p-mimic. (l) Western blot assay of the protein expression of E-cadherin, N-cadherin, and Vimentin in C33A cells after treatment with EV-miR-146a-5p-mimic. The data in the Figure were measurement data and expressed as mean ± standard deviation. The data between two groups were analyzed by unpaired *t*-test, and the comparison among multiple groups was performed by a one-way analysis of variance (Dunnett's post hoc test). ^*∗*^*p* < 0.05. Cell experiments were repeated 3 times independently.

To discern the impact of EVs on cervical cancer metastasis, we separated EVs from the supernatant medium of HeLa, CaSki, SiHa, and C33A cells as well as End1/E6E7 and HcerEpic cells, and observed the morphology of the separated particles under an electron microscope. As shown in Figures 2(a) and 2(b), the isolated EVs from CaSki and HcerEpic cells were basically uniform round-shaped or elliptical-shaped, with the diameter in the range of 30–120 nm determined by dynamic light scattering. Western blot assay results showed that the isolated vesicles were positive for EVs markers CD63, CD81, and TSG101 but negative for endoplasmic reticulum marker protein Calnexin, validating the successful extraction of EVs (Figure 2(c)). Next, RT-qPCR results unfolded that compared with EVs secreted by normal cervical epithelial cells, miR-146a-5p was expressed higher in EVs secreted by cervical cancer cells (Figure 2(d)). To further identify whether the high expression of miR-146a-5p in EVs secreted by cervical cancer cells is due to the increase of EVs secreted by cervical cancer cells or more miR-146a-5p encapsulated by EVs, we compared the expression of miR-146a-5p in the equal number of EVs isolated from cervical cancer cell line (CaSki) and normal cervical epithelial cell line (HcerEpic) and the expression of corresponding target genes in the isolated EVs. Western blot assay results described that the expression of CD63, CD81, and CD9 in EVs isolated from CaSki cells was the same as that in the EVs isolated from HcerEpic cells (Supplementary Figure 2F), indicating that the number of counted EVs was the same. Further RT-qPCR detection results revealed increase of miR-146a-5p in the EVs isolated from CaSki cells than that in the EVs isolated from HcerEpic cells (Supplementary Figure 2G), indicating that miR-146a-5p was more packaged into EVs isolated from CaSki cells than the EVs from HcerEpic cells after equalizing the EV count.

Next, the EVs secreted by the highly metastatic CaSki cells were extracted, and labeled with PKH67, and then co-cultured with the low-metastatic C33A cells for 30 and 120 minutes. The uptake of EVs by C33A cells was observed under a confocal fluorescence microscope. The results showed that EVs secreted by CaSki cells were internalized by C33A cells, which occurred at 30 minutes of co-culture (Figure 2(e)). Then, co-culture system depicted that C33A cells co-cultured with CaSki cells transfected with miR-146a-5p-Cy3 emitted red fluorescence; however, no obvious red fluorescence was noted in the C33A cells co-cultured with CaSki cells co-treated with miR-146a-5p-Cy3 and GW4869 (Figure 2(f)), indicating that EVs from CaSki cells could deliver miR-146a-5p to C33A cells. Next, we extracted the EVs from CaSki cells overexpressing miR-146a-5p. As described by RT-qPCR, an enhancement in the miR-146a-5p expression was witnessed in EVs from CaSki cells overexpressing miR-146a-5p while no significant difference occurred between the EVs from control cells and mimic–NC–treated cells (Figure 2(g)). These data implied that the overexpression of miR-146a-5p may lead to more miR-146a-5p packaged into EVs. C33A cells were co-incubated with EVs secreted by CaSki cells overexpressing miR-146a-5p (EV-miR-146a-5p mimic). Compared with that in the cells cultured with PBS, the expression of miR-146a-5p was increased in the C33A cells cultured with CaSki-secreted EVs (EV-control); while it was further increased following co-culture of EV-miR-146a-5p mimic (Figure 2(h)). Transwell assay showed that treatment with EV-control resulted in increased migrated and invaded C33A cells, while treatment with EV-miR-146a-5p-mimic caused an enhanced promotion (Figure 2(i), Supplementary Figure 1C). At the same time, we also found that EV-control weakened extracellular matrix adhesion and lowered the expression of E-cadherin but enhanced the apoptosis resistance ability and expression of N-cadherin and Vimentin in C33A cells; whereas, EV-miR-146a-5p-mimic caused consistent changes in larger folds (Figures 2(j)–2(l), Supplementary Figures 1D, 1G, and 1H). Therefore, miR-146a-5p carried by EVs secreted from high-metastatic cervical cancer cells could promote the malignancy of low-metastatic cervical cancer cells.

### 3.3. miR-146a-5p Could Negatively Target WWC2 to Activate the Hippo-YAP Signaling Pathway

**Figure 3:**
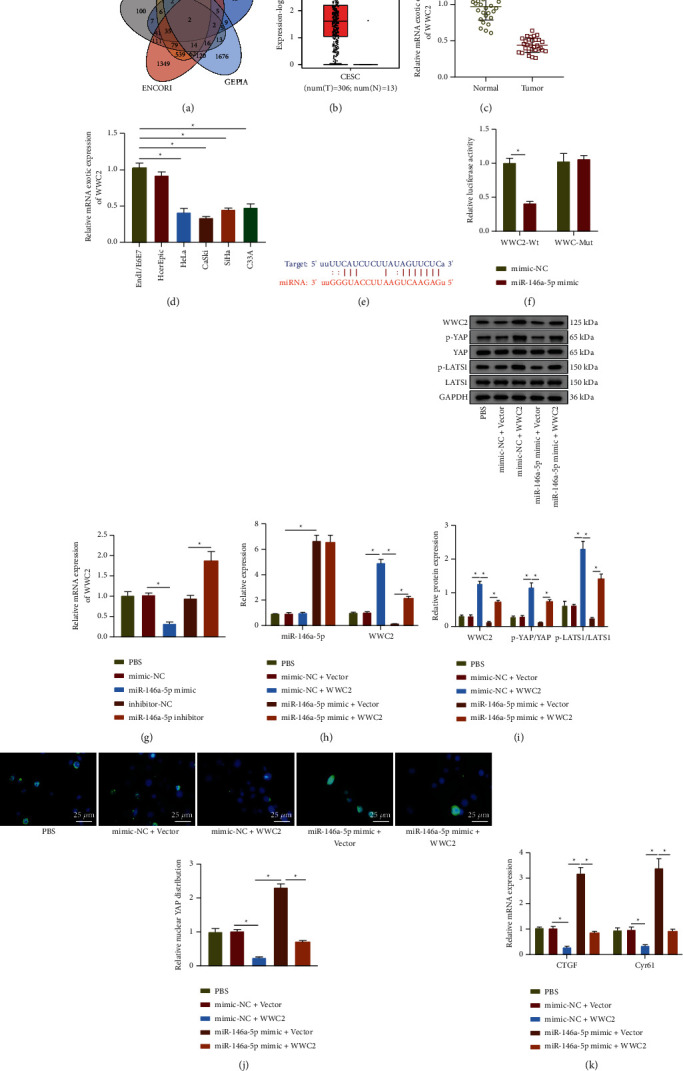
miR-146a-5p downregulates WWC2 to activate the Hippo-YAP signaling pathway. (a) The Venn map of the intersection of target genes obtained from the TargetScan, miRWalk, miRDB, and ENCORI databases and differentially expressed genes in GEPIA2 database in cervical cancer. (b) Analysis of the expression of WWC2 in cervical cancer by GEPIA2 database. Red represents cervical cancer samples, and gray represents normal samples, ^*∗*^*p* < 0.05. (c) RT-qPCR determination of WWC2 expression in 30 cases of human cervical cancer tissues and 30 cases of normal cervical tissues. (d) RT-qPCR determination of WWC2 expression in cervical cancer cell lines (HeLa, CaSki, SiHa, and C33A) and normal cervical epithelial cell lines (End1/E6E7 and HcerEpic). (e) The predicted binding site of miR-146a-5p and WWC2 from ENCORI database. (f) The dual luciferase reporter assay to identify the binding relationship between miR-146a-5p and WWC2. (g) RT-qPCR determination of WWC2 mRNA expression in C33A cells. (h) RT-qPCR detection of the expression of miR-146a-5p and WWC2 in C33A cells. (i) Western blot assay of the protein expression of WWC2, LATS1, and YAP, and the levels of LATS1 and YAP phosphorylation in C33A cells. (j) Immunofluorescence detection of YAP location in C33A cells, scale bar: 25 *μ*m. (k) RT-qPCR determination of the mRNA expression of CTGF and Cyr61 in C33A cells. The data in the Figure were measurement data and expressed as mean ± standard deviation. The data between two groups were analyzed by unpaired *t*-test, and the comparison among multiple groups was performed by a one-way analysis of variance (Dunnett's post hoc test). ^*∗*^*p* < 0.05. Cell experiments were repeated 3 times independently.

We then predicted the downstream targets of miR-146a-5p utilizing TargetScan, miRWalk, miRDB, and ENCORI databases, and used GEPIA database to find the differentially expressed genes (log2FC < −1.5, *p* < 0.01). Finally, two candidate genes were harvested after intersection: WWC2 and NOVA1 (Figure 3(a)). It has been reported that WWC2 gene can inhibit tumor metastasis [22], while NOVA1 usually plays an oncogenic role [23]. Therefore, we chose WWC2 as the target gene.  Figure 3(b) shows the WWC2 expression in cervical cancer samples in the TCGA database utilizing the GEPIA2 tool. RT-qPCR results consistently showed downregulation of WWC2 in cervical cancer clinical tissues and cell lines (Figures 3(c) and 3(d)).

Based on the binding site of miR-146a-5p and WWC2 shown by ENCORI database (Figure 3(e)), the WWC2-3′UTR-mut plasmid was constructed. The results of the dual luciferase reporter experiment described that the cells co-transfected with miR-146a-5p mimic and WWC2-WT had a decreased luciferase activity (Figure 3(f)). RT-qPCR results summarized that the WWC2 mRNA expression was decreased in the cells transfected with miR-146a-5p mimic but elevated in those transfected with miR-146a-5p inhibitor (Figure 3(g)). Hence, miR-146a-5p could limit WWC2 expression in cervical cancer cells.

The activity of the Hippo-YAP signaling pathway assayed by Western blot assay unfolded that the levels of LATS1 and YAP phosphorylation were increased after WWC2 was overexpressed, while those were diminished after overexpression of miR-146a-5p. Simultaneous overexpression of WWC2 and miR-146a-5p could reverse the inhibiting effect of single miR-146a-5p overexpression on the levels of LATS1 and YAP phosphorylation (Figures 3(h) and 3(i)). Next, immunofluorescence was performed to determine YAP nuclear localization. The results showed suppressed nuclear YAP expression after overexpression of WWC2. The nuclear YAP expression was increased after mimic of miR-146a-5p, which was diminished after restoration of WWC2 (Figure 3(j)). Furthermore, mRNA expression of Hippo-YAP signaling pathway downstream molecules (CTGF and Cyr61) in C33A cells was decreased after overexpression of WWC2, but elevated after overexpression of miR-146a-5p. However, overexpression of WWC2 reversed the promotion of CTGF and Cyr61 mRNA expression induced by mimic of miR-146a-5p (Figure 3(k)). In conclusion, miR-146a-5p activated the Hippo-YAP signaling pathway through targeting WWC2 in cervical cancer cells.

### 3.4. miR-146a-5p Carried by Cervical Cancer Cells-Secreted EVs Could Facilitate Cervical Cancer Metastasis through Downregulating WWC2

**Figure 4:**
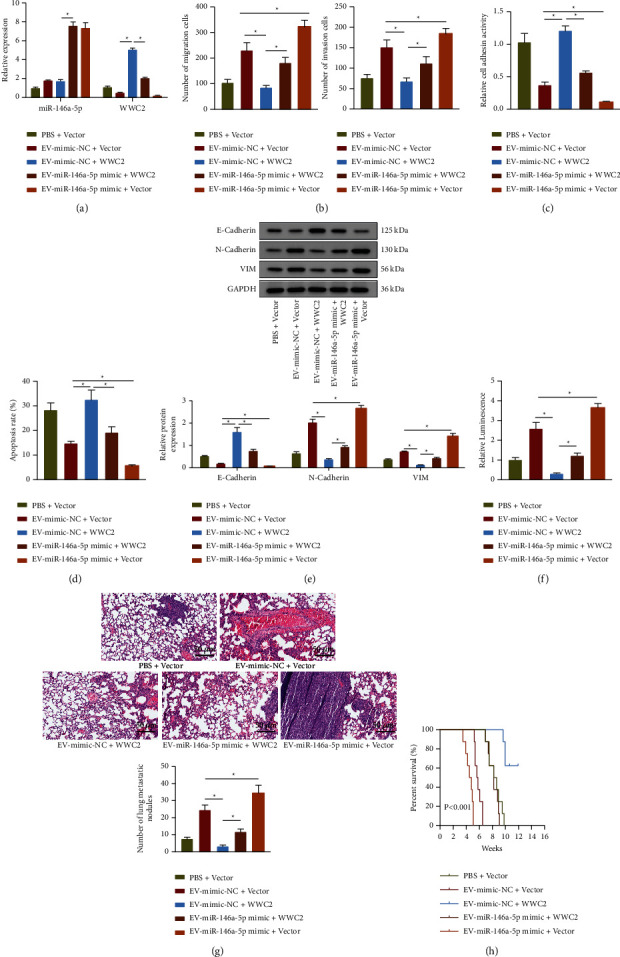
miR-146a-5p carried by EVs enhances cervical cancer metastasis through suppressing WWC2. (a) RT-qPCR determination of miR-146a-5p and WWC2 expression in C33A cells. (b) Transwell assay of the invasion and migration of C33A cells. (c) The adhesion of C33A cells to the extracellular matrix. (d) TUNEL staining of apoptosis of C33A cells. (e) Western blot assay of E-cadherin, N-cadherin, and Vimentin protein expression in C33A cells. (f) The *in vivo* fluorescence imaging of nude mice injected with 5 × 10^5^ stable luciferase-labeled C33A cells through the tail vein, *n* = 8. (g) H&E staining of lung tissues of nude mice and the number of metastatic nodes in the lung, scale bar: 50 *μ*m. (h) Survival curve of nude mice, *n* = 8. The data in the Figure were measurement data and expressed as mean ± standard deviation. The comparison among multiple groups was performed by a one-way analysis of variance (Dunnett's post hoc test). ^*∗*^*p* < 0.05. Cell experiments were repeated 3 times independently.

To explore whether miR-146a-5p mediated WWC2 to affect cervical cancer metastasis, C33A cells overexpressing WWC2 were co-cultured with EVs secreted by the CaSki cells treated with mimic-NC or miR-146a-5p mimic (EV-mimic-NC or EV-miR-146a-5p mimic). RT-qPCR results showed that the WWC2 expression was elevated by treatment with WWC2 overexpression plasmid in the C33A cells cultured with EV-mimic-NC. The expression of miR-146a-5p was increased while that of WWC2 was decreased by co-culture with EV-miR-146a-5p mimic in either the C33A cells treated with Vector or C33A cells overexpressing WWC2 (Figure 4(a)). These results summarized that miR-146a-5p carried by EVs could diminish WWC2 expression. Furthermore, our results demonstrated that WWC2 overexpression reduced the numbers of migrated and invaded C33A cells cultured with EV-mimic-NC, which was reversed by the delivery of more miR-146a-5p *via* EVs. Additionally, an increase was noted in the numbers of migrated and invaded C33A cells treated with EV-miR-146a-5p mimic + Vector relative to those in cells treated with EV-mimic-NC + Vector (Figure 4(b)). At the same time, WWC2 overexpression resulted in promoted extracellular matrix adhesion ability and elevated expression of E-cadherin, and weakened anti-anoikis ability and decreased expression of N-cadherin and Vimentin in the C33A cells cultured with EV-mimic-NC. However, EV-miR-146a-5p mimic could reverse the effects induced by WWC2 overexpression (Figures 4(c)–4(e)). Furthermore, nude mice lung metastasis models were established for *in vivo* validation. The results of *in vivo* imaging, H&E staining, and the curve for mouse survival showed that lung metastasis of the mice injected with EV-mimic-NC was suppressed and their survival time was increased upon WWC2 overexpression, whereas the lung metastasis in the mice overexpressing WWC2 was enhanced by treatment with EV-miR-146a-5p mimic, corresponding to shortened survival time. Treatment with EV-miR-146a-5p mimic + Vector enhanced the lung metastasis of the mice while arresting mouse survival time (Figures 4(f)–4(h)). Taken together, miR-146a-5p carried by EVs accelerated the metastasis of cervical cancer by inhibiting WWC2.

### 3.5. WWC2 Regulated Actin Dynamics to Limit the Metastasis of Cervical Cancer Cells through the Hippo-YAP Pathway

**Figure 5:**
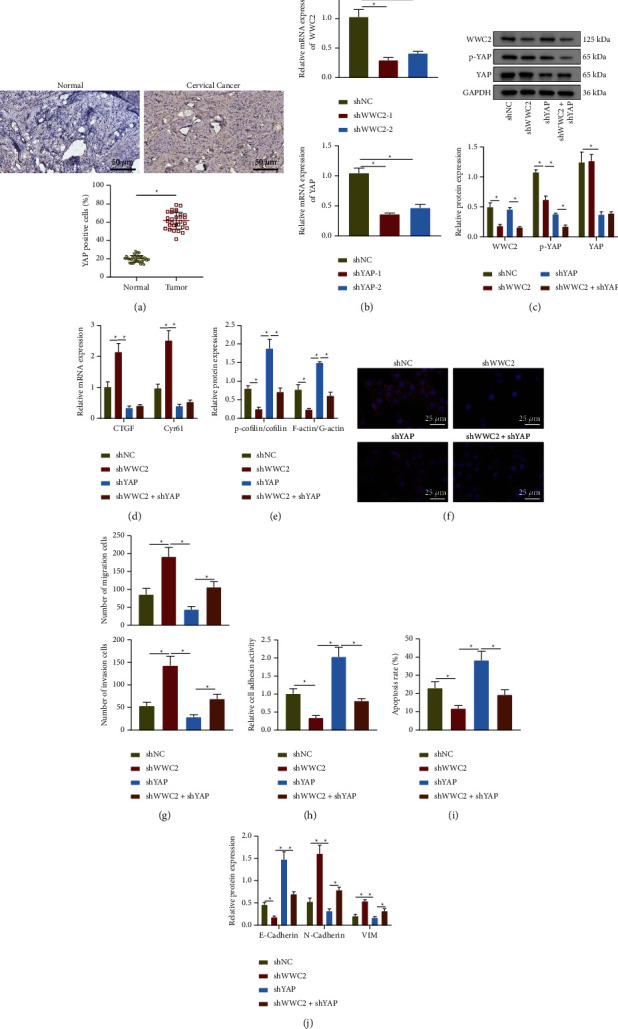
WWC2 regulates actin dynamics and impedes cervical cancer metastasis through the Hippo-YAP pathway. (a) Immunohistochemical detection of YAP expression in human cervical cancer tissues and normal cervical tissues, scale bar: 50 *μ*m. (b) RT-qPCR determination of WWC2 and YAP mRNA expression in C33A cells after WWC2 or YAP silencing. (c) Western blot assay of WWC2 and YAP expression and the level of YAP phosphorylation in C33A cells after WWC2 or YAP silencing. (d) RT-qPCR determination of CTGF and Cyr61 mRNA expression in C33A cells after WWC2 or YAP silencing. (e) Western blot assay of cofilin, p-cofilin, F-actin, and G-actin expression in C33A cells after WWC2 or YAP silencing. (f) Phalloidin staining of the cytoskeleton of C33A cells after WWC2 or YAP silencing, scale bar: 25 *μ*m. (g) Transwell assay of the invasion and migration of C33A cells after WWC2 or YAP silencing. (h) The adhesion of C33A cells to the extracellular matrix after WWC2 or YAP silencing. (i) TUNEL staining for apoptosis of C33A cells after WWC2 or YAP silencing. (j) Western blot assay of E-cadherin, N-cadherin, and Vimentin protein expression in C33A cells after WWC2 or YAP silencing. The data in the figure were measurement data and expressed as mean ± standard deviation. The comparison among multiple groups was performed by a one-way analysis of variance (Dunnett's post hoc test). ^*∗*^*p* < 0.05. Cell experiments were repeated 3 times independently.

YAP can promote activity of the actin depolymerization factor cofilin by inhibiting the phosphorylation of cofilin, thereby changing the dynamics of F-actin/G-actin and accelerating cancer metastasis [17, 24]. Expression and location of YAP in clinical cervical cancer samples assayed utilizing immunohistochemical staining described increased protein expression of YAP and elevated nuclear expression in cervical cancer tissues (Figure 5(a)). Next, shRNAs against WWC2 and YAP were constructed to silence WWC2 and YAP, of which shWWC2-1 and shYAP-1 had better silencing efficacy and were thus selected for subsequent experiments (Figure 5(b)). Consistently, Western blot assay confirmed the silencing efficacy (Figure 5(c)). In addition, RT-qPCR results revealed that CTGF and Cyr61 were downregulated after YAP knockdown, while concomitant silencing of WWC2 and YAP could also reduce the mRNA expression of CTGF and Cyr61 (Figure 5(d)). Western blot assay results exhibited that the phosphorylation of cofilin and the ratio of F-actin/G-actin were both decreased after WWC2 knockdown. YAP knockdown led to enhanced cofilin phosphorylation and F-actin/G-actin ratio, which were both diminished after knocking WWC2 down (Figure 5(e)).

Immunofluorescence displayed that the stress fibers were shortened and weakened upon knockdown of WWC2, and the stress fibers were elongated after knocking YAP down, with the presence of actin filaments. Concomitant silencing of WWC2 and YAP negated the effects of YAP silencing alone on the cytoskeleton (Figure 5(f)). The above-mentioned results suggested that WWC2 promoted phosphorylation of actin depolymerization factor cofilin through YAP and inhibit its activity, thereby inhibiting the depolymerization of F-actin to G-actin in cervical cancer cells.

Moreover, loss of YAP suppressed the migrated and invaded abilities of C33A cells while loss of WWC2 enhanced these abilities. WWC2 silencing counteracted the anti-migratory and anti-invasive effects of YAP silencing (Figure 5(g)). At the same time, we also found that the ability to adhere to the extracellular matrix and the expression of E-cadherin were enhanced, while the anti-anoikis ability and N-cadherin and Vimentin expression were suppressed after YAP was knocked down alone in C33A cells; silencing WWC2 caused weakened extracellular matrix adhesion ability, decreased E-cadherin expression, enhanced anti-anoikis ability, and increased expression of N-cadherin and Vimentin. These changes induced by YAP silencing were reversed by WWC2 silencing (Figures 5(h)–5(j)). Collectively, WWC2 suppressed cervical cancer metastasis through blocking the Hippo-YAP pathway.

### 3.6. miR-146a-5p Carried by Cervical Cancer Cells-Secreted EVs Accelerated the Metastasis of Cervical Cancer through Regulating the WWC2/YAP-Mediated Actin Dynamics

**Figure 6:**
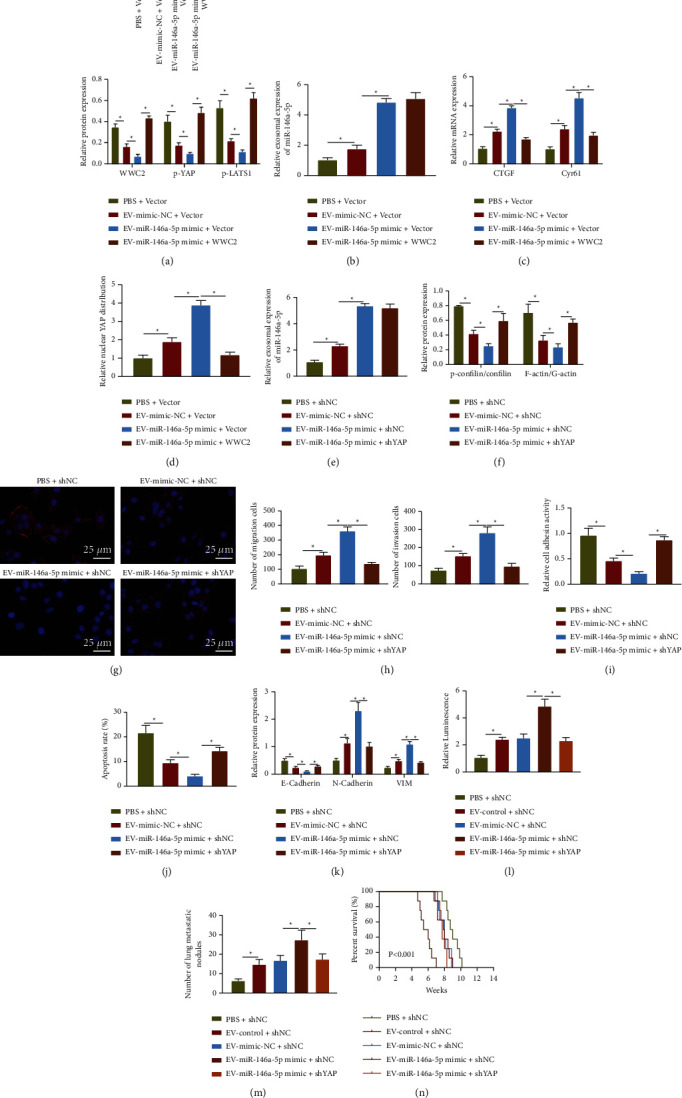
miR-146a-5p carried by EVs activates the Hippo-YAP pathway through targeting WWC2 to facilitate cervical cancer metastasis. (a) Western blot assay of WWC2 protein and the levels of LATS1 and YAP phosphorylation in C33A cells. (b) RT-qPCR determination of miR-146a-5p expression in C33A cells. (c) RT-qPCR determination of CTGF and Cyr61 mRNA expression in C33A cells. (d) Immunofluorescence detection of YAP location in C33A cells. (e) RT-qPCR determination of miR-146a-5p in C33A cells. (f) Western blot assay of YAP, cofilin, p-cofilin, F-actin, and G-actin expression in C33A cells. (g) Phalloidin staining of the cytoskeleton of C33A cells, scale bar: 25 *μ*m. (h) Transwell assay of the invasion and migration of C33A cells. (i) Adhesion of C33A cells to the extracellular matrix. (j) TUNEL staining of C33A cell apoptosis. (k) Western blot assay of the protein expression of E-cadherin, N-cadherin, and Vimentin in C33A cells. (l) The *in viv*o fluorescence imaging of nude mice injected with 5 × 10^5^ stable luciferase-labeled C33A cells through tail vein, *n* = 8. (m) The metastatic nodes in the lunge of nude mice, *n* = 8. (n) Survival curve of nude mice, *n* = 8. The data in the figure were measurement data and expressed as mean ± standard deviation. The comparison among multiple groups was performed by a one-way analysis of variance (Dunnett's post hoc test). ^*∗*^*p* < 0.05. Cell experiments were repeated 3 times independently.

To identify whether miR-146a-5p carried by EVs affected actin dynamics through WWC2/YAP, C33A cells were treated with Vector and WWC2, and then cultured with CaSki cells-secreted EVs. Western blot results showed that EV-mimic-NC or EV-miR-146a-5p mimic downregulated WWC2, and the levels of LATS1 and YAP phosphorylation in the C33A cells. On the contrary, WWC2 overexpression rescued the levels of LATS1 and YAP phosphorylation inhibited by EV-miR-146a-5p mimic (Figure 6(a)). RT-qPCR results showed that EV-mimic-NC or EV-miR-146a-5p mimic elevated the expression of miR-146a-5p, CTGF, and Cyr61 in the C33A cells; whereas, WWC2 overexpression reversed the effect of EV-miR-146a-5p mimic on CTGF and Cyr61 expression (Figures 6(b) and 6(c)). At the same time, EV-mimic-NC or EV-miR-146a-5p mimic elevated nuclear YAP expression in C33A cells, but the nuclear YAP expression enhanced by EV-miR-146a-5p mimic was diminished after WWC2 overexpression (Figure 6(d)). Therefore, miR-146a-5p carried by cervical cancer cells-secreted EVs activated the Hippo-YAP pathway through inhibition of WWC2. Next, the stably transduced C33A cells (shNC and shYAP) were cultured with EV-mimic-NC or EV-miR-146a-5p mimic. Western blot assay and immunofluorescence detection showed that the phosphorylation of cofilin and the ratio of F-actin/G-actin were decreased, and the stress fibers were shortened and weakened in the C33A cells treated with shNC after culture with EV-mimic-NC or EV-miR-146a-5p mimic. The changes caused by EV-miR-146a-5p mimic were reversed after YAP silencing (Figures 6(e)–6(g)).

Additionally, YAP silencing suppressed the migration and invasion (Figure 6(h)), enhanced adhesion potential to extracellular matrix and E-cadherin expression, and weakened anti-anoikis and N-cadherin and Vimentin expression (Figures 6(i)–6(k)) in C33A cells mediated by EV-miR-146a-5p mimic. Meanwhile, *in vivo* data suggested that the YAP knockdown suppressed lung metastasis, and increased survival time of mice treated with EV-miR-146a-5p mimic (Figures 6(l)–6(n)). All in all, miR-146a-5p delivered by EVs contributed to cervical cancer metastasis through WWC2-mediated Hippo-YAP pathway.

## 4. Discussion

Chemotherapy and radiotherapy are the common therapeutic treatments of cervical cancer but alternative therapies are urgently required due to their side effects and toxicity [25]. Therefore, extensive studies of the molecular mechanisms that regulate the metastatic potential of cervical cancer cells are required to seek new therapeutic targets and strategies for this malignancy. In this study, we provided evidence that the miR-146a-5p carried by cervical cancer cells-derived EVs activated the Hippo-YAP signaling pathway through WWC2 to affect actin dynamics and promote cervical cancer metastasis.

Our study discerned that miR-146a-5p was elevated in the cervical cancer tissues. It is reported that cancer-derived EVs carry various tumor-derived molecules such as mutated DNA and RNA fragments, miRNA, and protein [7]. Consistent with our finding, a prior study has reported an elevated miR-146a expression in cervical intraepithelial neoplasia and cervical cancer [25], which is also demonstrated in a recent study, suggesting the diagnostic value of miR-146a-5p for cervical cancer [11]. The tumor-promotive role of miR-146a in cervical cancer has been unraveled in increasing studies; for instance, miR-146a can enhance cancer cell proliferation [26]. miR-146a possesses potential to promote cervical cancer cell viability through modulation of IRAK1 and TRAF6 [10]. In addition to the pro-proliferative role of miR-146a-5p, this study further demonstrated that mimic of miR-146a-5p could strengthen the anti-anoikis ability and drive the EMT process. Furthermore, evidence has been presented demonstrating an enrichment of miR-146a-5p in the EVs secreted by cervical cancer cells. Similarly, miR-146a-5p in EVs from cancer-associated fibroblasts can enhance EMT to accelerate prostate cancer cell metastasis [27]. In addition, it is reported that breast cancer-derived EVs carrying miR-146a contributed to the invasion and metastasis of breast cancer cells through activating cancer-associated fibroblasts [28].

In this experiment, the results presented that miR-146a-5p could target WWC2 and inhibited its expression to activate the Hippo-YAP signaling pathway. WWC2 is shown as an anti-oncogene in human cancers that can be mediated by tumor-promotive miRNAs. Similar with our findings, downregulation of WWC2 by miR-21-5p contributes to progression of lung adenocarcinoma [13], while upregulation of WWC2 caused by loss of miR-10a reverses the induction of EMT in pancreatic cancer stem cells [14]. Furthermore, WWC2 inhibits the metastasis of hepatocellular carcinoma by negatively modulating the Hippo pathway [22]. Our data also showed that WWC2 phosphorylated the transcriptional co-activator YAP to promote the phosphorylation of the actin depolymerization factor cofilin, whereby promoting the depolymerization of F-actin to G-actin. A prior study has reported the importance of dynamic rearrangement of F-actin in presenting the therapeutic effects of antitumor agents in tumor cells [29]. Consistent with our findings, over-expressing YAP leads to cytoskeletal rearrangement through regulating the dynamics of F-actin/G-actin turnover, thus accelerating the migration of gastric cancer cells [17]. In the *in vivo* tumor metastatic model, we also demonstrated that cervical cancer cells-secreted EVs carrying miR-146a-5p promoted the metastasis of cervical cancer through activating YAP.

## 5. Conclusions

**Figure 7:**
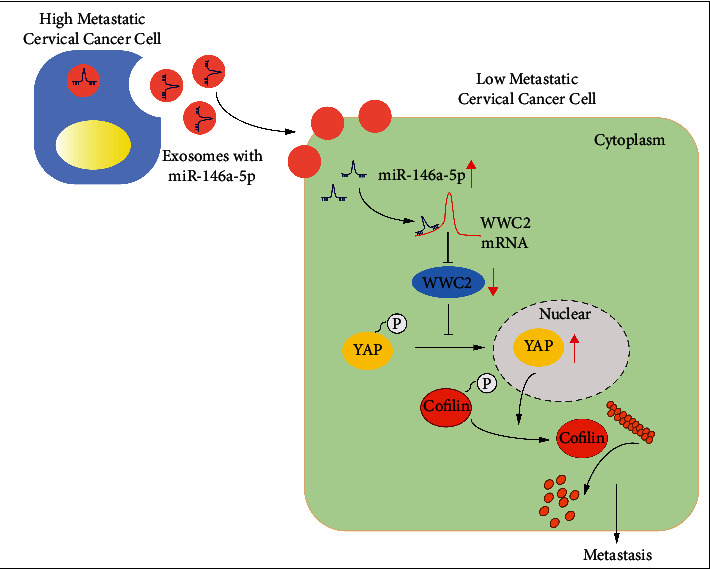
Molecular mechanism diagram illustrating the transfer of miR-146a-5p *via* cervical cancer cells-secreted EVs to promote the metastasis of cervical cancer. EVs secreted by high-metastatic cervical cancer cells carrying miR-146a-5p can be internalized by low-metastatic cervical cancer cells, and then miR-146a-5p inhibited WWC2 expression to promote dephosphorylation and nuclear translocation of YAP. Consequently, YAP promoted the activity of actin depolymerization factor cofilin by inhibiting its phosphorylation, thereby changing the dynamics of F-actin/G-actin and leading to metastasis of low-metastatic cervical cancer cells.

Conclusively, our study showed evidence that EVs secreted by high-metastatic cervical cancer cells transmitted miR-146a-5p into low-metastatic cervical cancer cells, which activated the WWC2-mediated Hippo-YAP signaling pathway and altered the dynamics of F-actin/G-actin, finally leading to cancer metastasis (Figure 7). This study mainly highlights a promising preventive strategy based on cancer-derived EVs against cancer metastasis. However, due to the small clinical sample size in the study, further larger-scale verification is required in the future.

## Data Availability

The datasets generated/analyzed during the current study are available from the corresponding author upon request.

## Supplementary Materials

Supplementary Materials Supplementary Figure 1 A, Representative image of Figure 1H. B, Representative image of Figure 1I. C, Representative image of Figure 2I. D, Representative image of Figure 2J. E, Representative image of Figure 1J. F, Representative image of Figure 1K. G, Representative image of Figure 2K. H, Representative image of Figure 2L. Supplementary Figure 2 Effect of miR-146a-5p on the invasion, migration, apoptosis, and EMT of HcerEpic cells. A, RT-qPCR determination of miR-146a-5p expression in HcerEpic cells transfected with miR-146a-5p mimic. B, Transwell assay of the migration and invasion of HcerEpic cells transfected with miR-146a-5p mimic. C, The adhesion of HcerEpic cells to the extracellular matrix after miR-146a-5p enhancement. D, Apoptosis of HcerEpic cells transfected with miR-146a-5p mimic determined by TUNEL assay. E, Western blot assay of EMT marker proteins E-cadherin, N-cadherin, and Vimentin in HcerEpic cells transfected with miR-146a-5p mimic. F, Western blot assay of CD63, CD81, and CD9 proteins in the EVs isolated from HcerEpic cells or CaSki cells. G, RT-qPCR determination of miR-146a-5p expression in the EVs isolated from HcerEpic cells or CaSki cells. The data in the figure were measurement data and expressed as mean ± standard deviation. The data between multiple groups were analyzed by a one-way analysis of variance (Dunnett's post hoc test). ^*∗*^*p* < 0.05. Cell experiments were repeated 3 times independently. Supplementary Figure 3 Effect of hsa-miR-7-5p and hsa-miR-20b-5p on the biological behavior of cervical cancer cells. A, RT-qPCR determination of hsa-miR-7-5p and hsa-miR-20b-5p expression in 30 cases of human cervical cancer tissues and 30 cases of normal cervical tissues. B, RT-qPCR determination of the expression of hsa-miR-7-5p and hsa-miR-20b-5p in cervical cancer cell lines (HeLa, CaSki, SiHa, and C33A) and normal cervical epithelial cell lines (End1/E6E7 and HcerEpic). C, RT-qPCR determination of the expression of hsa-miR-7-5p and hsa-miR-20b-5p in C33A cells transfected with hsa-miR-7-5p mimic, hsa-miR-20b-5p mimic, hsa-miR-7-5p inhibitor, or hsa-miR-20b-5p inhibitor. D, E, Transwell assay of the invasion and migration of C33A cells transfected with hsa-miR-7-5p mimic, hsa-miR-20b-5p mimic, hsa-miR-7-5p inhibitor, or hsa-miR-20b-5p inhibitor. F, The adhesion of C33A cells to the extracellular matrix following transfection with hsa-miR-7-5p mimic, hsa-miR-20b-5p mimic, hsa-miR-7-5p inhibitor, or hsa-miR-20b-5p inhibitor. G, The apoptosis of C33A cells transfected with hsa-miR-7-5p mimic, hsa-miR-20b-5p mimic, hsa-miR-7-5p inhibitor, or hsa-miR-20b-5p inhibitor assessed by TUNEL staining. The data in the figure were measurement data and expressed as mean ± standard deviation. The data between multiple groups were analyzed by a one-way analysis of variance (Dunnett's post hoc test). ^*∗*^*p* < 0.05. Cell experiments were repeated 3 times independently. Supplementary Table 1 shRNA sequences. Supplementary Table 2 Primer sequences for RT-qPCR. Note: RT-qPCR, reverse transcription-quantitative polymerase chain reaction; F, forward; R, reverse; miR-146a-5p, microRNA-146a-5p; WWC2, WW, and C2 domain containing 2; YAP, Yes-associated protein; CTGF, connective tissue growth factor; GAPDH, glyceraldehyde-3-phosphate dehydrogenase.
