# Supplementary material for: Cervical Cancer Cells-Derived Extracellular Vesicles Containing microRNA-146a-5p Affect Actin Dynamics to Promote Cervical Cancer Metastasis by Activating the Hippo-YAP Signaling Pathway via WWC2
Source: J Oncol. 2022 Jun 28;2022:4499876. doi: 10.1155/2022/4499876 (PMC9256433; doi:10.1155/2022/4499876)
Supplement: Supplementary Materials — Supplementary Figure 1 A, Representative image of Figure 1H. B, Representative image of Figure 1I. C, Representative image of Figure 2I. D, Representative image of Figure 2J. E, Representative image of Figure 1J. F, Representative image of Figure 1K. G, Representative image of Figure 2K. H, Representative image of Figure 2L. Supplementary Figure 2 Effect of miR-146a-5p on the invasion, migration, apoptosis, and EMT of HcerEpic cells. A, RT-qPCR determination of miR-146a-5p expression in HcerEpic cells transfected with miR-146a-5p mimic. B, Transwell assay of the migration and invasion of HcerEpic cells transfected with miR-146a-5p mimic. C, The adhesion of HcerEpic cells to the extracellular matrix after miR-146a-5p enhancement. D, Apoptosis of HcerEpic cells transfected with miR-146a-5p mimic determined by TUNEL assay. E, Western blot assay of EMT marker proteins E-cadherin, N-cadherin, and Vimentin in HcerEpic cells transfected with miR-146a-5p mimic. F, Western blot assay of CD63, CD81, and CD9 proteins in the EVs isolated from HcerEpic cells or CaSki cells. G, RT-qPCR determination of miR-146a-5p expression in the EVs isolated from HcerEpic cells or CaSki cells. The data in the figure were measurement data and expressed as mean ± standard deviation. The data between multiple groups were analyzed by a one-way analysis of variance (Dunnett's post hoc test). ∗p < 0.05. Cell experiments were repeated 3 times independently. Supplementary Figure 3 Effect of hsa-miR-7-5p and hsa-miR-20b-5p on the biological behavior of cervical cancer cells. A, RT-qPCR determination of hsa-miR-7-5p and hsa-miR-20b-5p expression in 30 cases of human cervical cancer tissues and 30 cases of normal cervical tissues. B, RT-qPCR determination of the expression of hsa-miR-7-5p and hsa-miR-20b-5p in cervical cancer cell lines (HeLa, CaSki, SiHa, and C33A) and normal cervical epithelial cell lines (End1/E6E7 and HcerEpic). C, RT-qPCR determination of the expression of hsa-miR-7-5p and hsa-miR-2 [file 4499876.f1.zip › 4499876.f1/Supplementary Figures.docx]

**
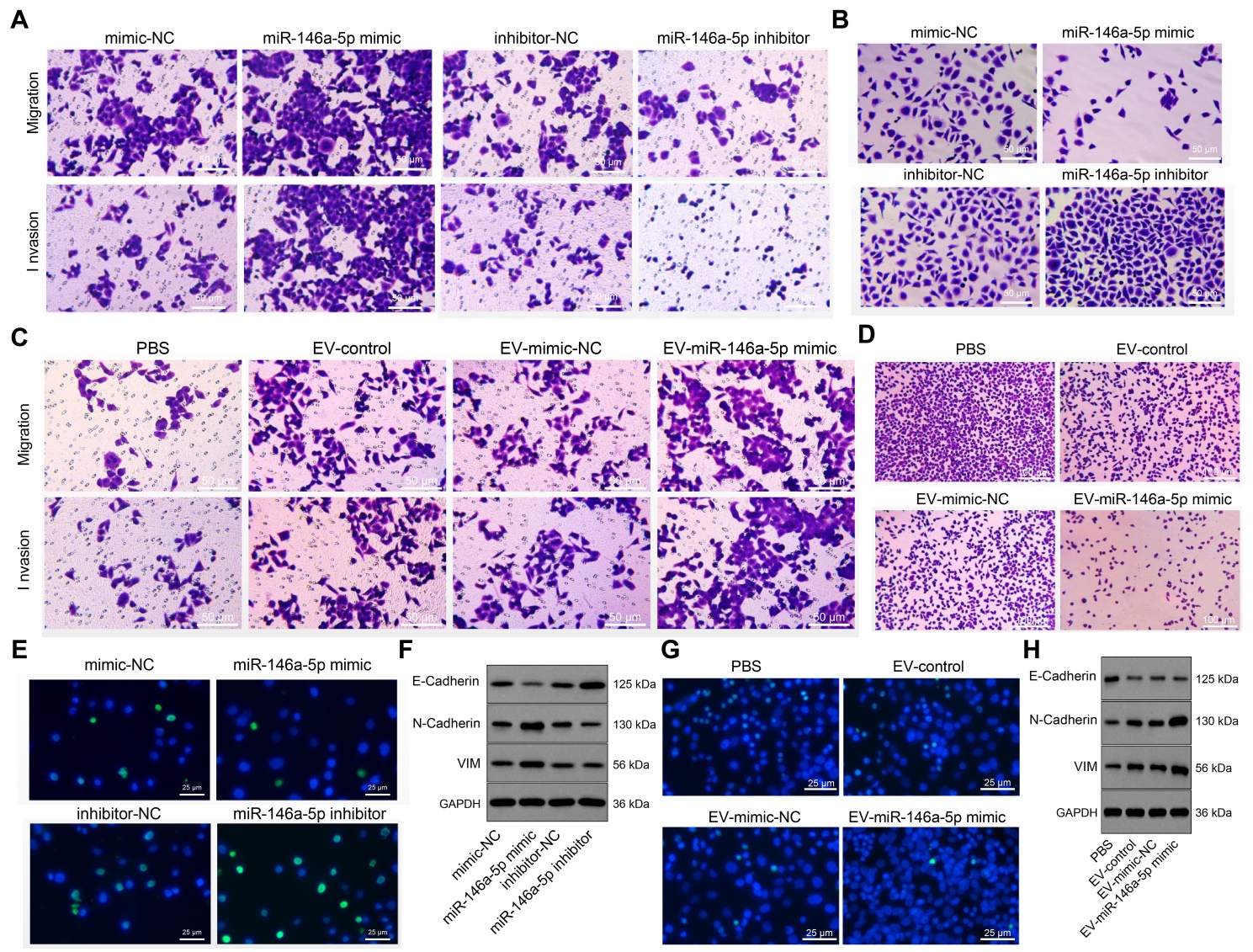
**

**Supplementary Figure. 1** A, Representative image of figure 1H. B, Representative image of figure 1I. C, Representative image of figure 2I. D, Representative image of figure 2J. E, Representative image of figure 1J. F, Representative image of figure 1K. G, Representative image of figure 2K. H, Representative image of figure 2L.

**
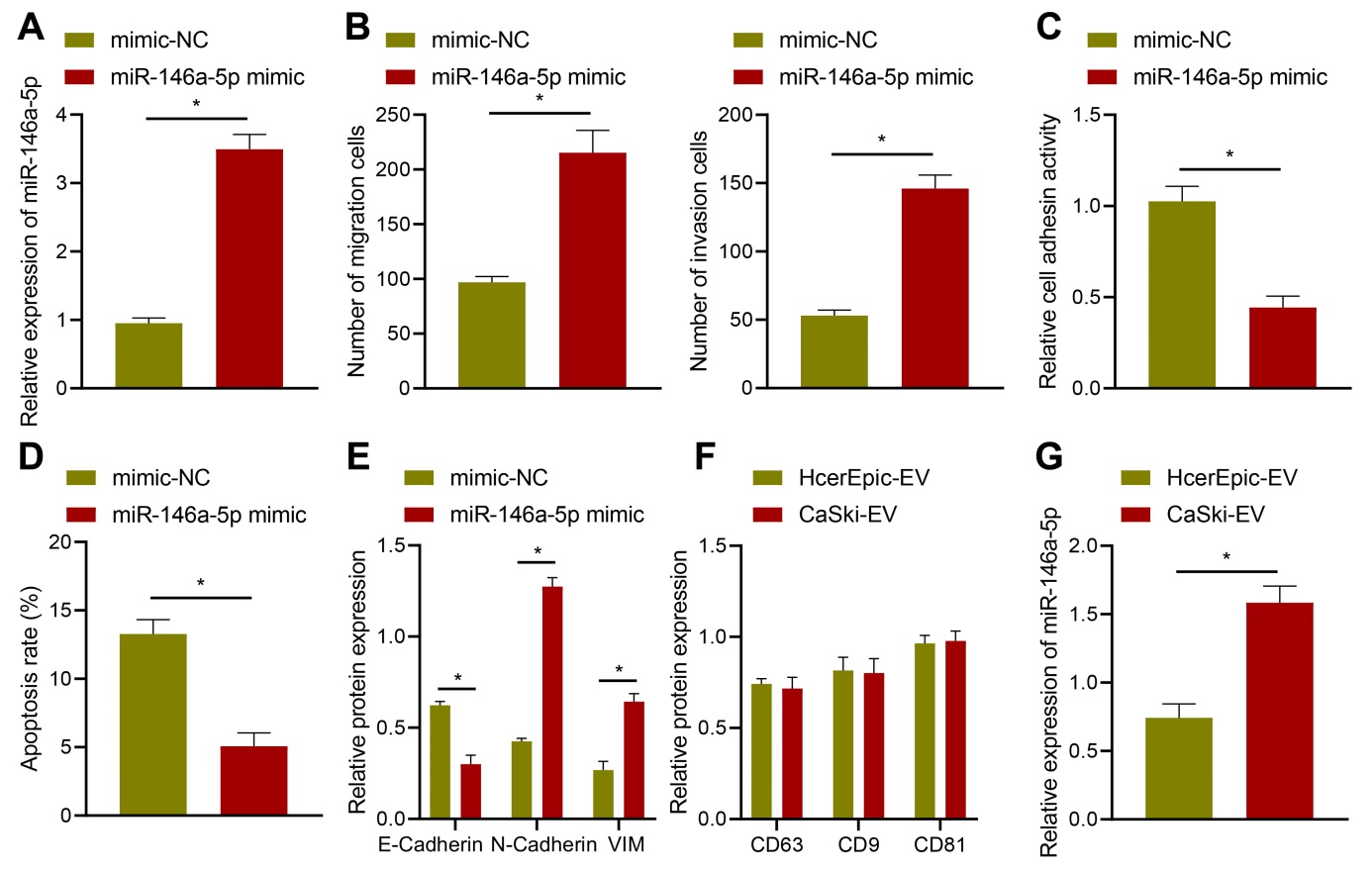
**

**Supplementary Figure. 2** Effect of miR-146a-5p on the invasion, migration, apoptosis and EMT of HcerEpic cells. A, RT-qPCR determination of miR-146a-5p expression in HcerEpic cells transfected with miR-146a-5p mimic. B, Transwell assay of the migration and invasion of HcerEpic cells transfected with miR-146a-5p mimic. C, The adhesion of HcerEpic cells to the extracellular matrix after miR-146a-5p enhancement. D, Apoptosis of HcerEpic cells transfected with miR-146a-5p mimic determined by TUNEL assay. E, Western blot assay of EMT marker proteins E-cadherin, N-cadherin and Vimentin in HcerEpic cells transfected with miR-146a-5p mimic. F, Western blot assay of CD63, CD81 and CD9 proteins in the EVs isolated from HcerEpic cells or CaSki cells. G, RT-qPCR determination of miR-146a-5p expression in the EVs isolated from HcerEpic cells or CaSki cells. The data in the figure were measurement data and expressed as mean ± standard deviation. The data between multiple groups were analyzed by a one-way analysis of variance (Dunnett’s post hoc test). * *p* < 0.05. Cell experiments were repeated 3 times independently.

**
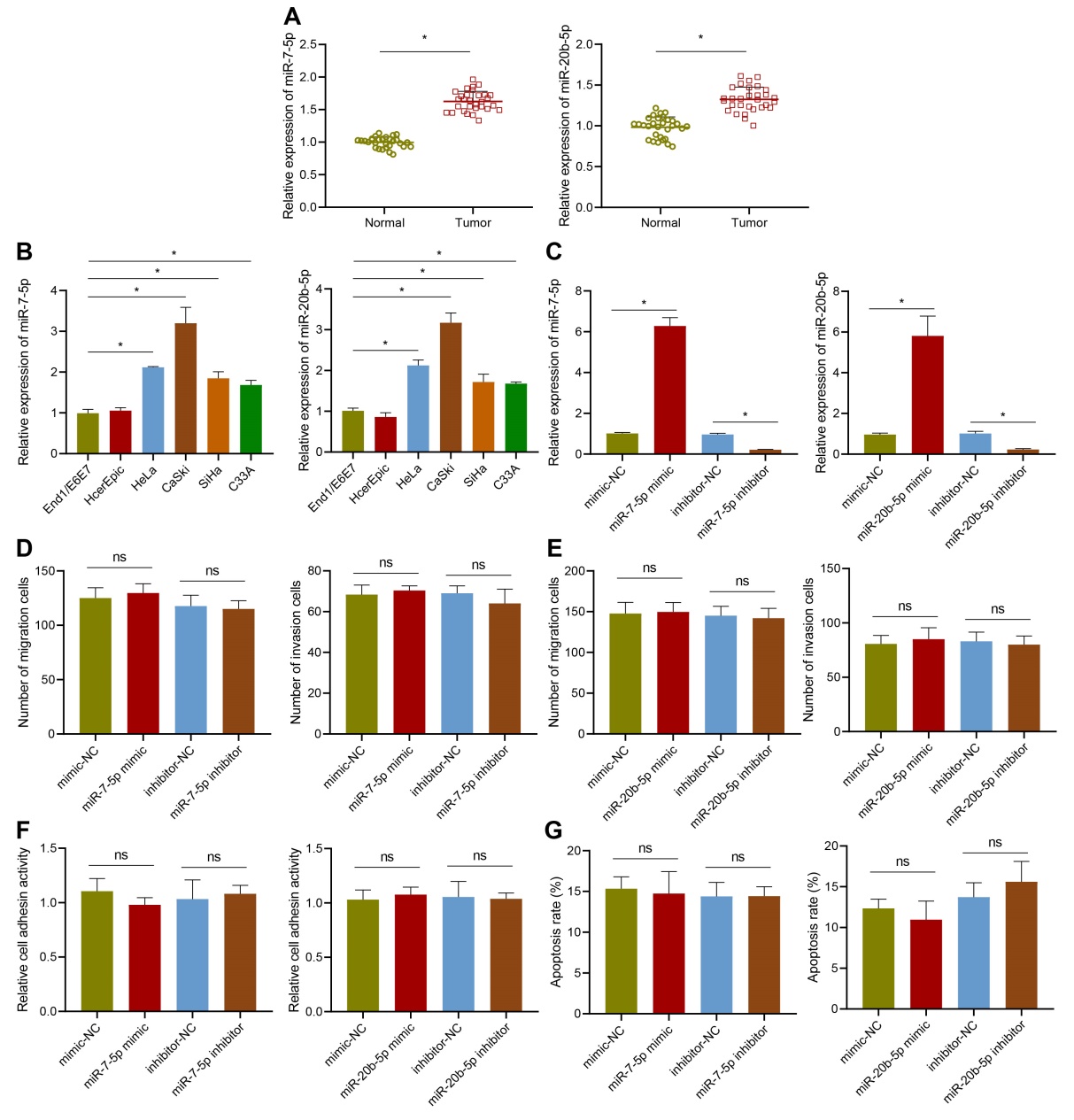
**

**Supplementary Figure. 3** Effect of hsa-miR-7-5p and hsa-miR-20b-5p on the biological behavior of cervical cancer cells. A, RT-qPCR determination of hsa-miR-7-5p and hsa-miR-20b-5p expression in 30 cases of human cervical cancer tissues and 30 cases of normal cervical tissues. B, RT-qPCR determination of the expression of hsa-miR-7-5p and hsa-miR-20b-5p in cervical cancer cell lines (HeLa, CaSki, SiHa, and C33A) and normal cervical epithelial cell lines (End1/E6E7 and HcerEpic). C, RT-qPCR determination of the expression of hsa-miR-7-5p and hsa-miR-20b-5p in C33A cells transfected with hsa-miR-7-5p mimic, hsa-miR-20b-5p mimic, hsa-miR-7-5p inhibitor or hsa-miR-20b-5p inhibitor. D, E, Transwell assay of the invasion and migration of C33A cells transfected with hsa-miR-7-5p mimic, hsa-miR-20b-5p mimic, hsa-miR-7-5p inhibitor or hsa-miR-20b-5p inhibitor. F, The adhesion of C33A cells to the extracellular matrix following transfection with hsa-miR-7-5p mimic, hsa-miR-20b-5p mimic, hsa-miR-7-5p inhibitor or hsa-miR-20b-5p inhibitor. G, The apoptosis of C33A cells transfected with hsa-miR-7-5p mimic, hsa-miR-20b-5p mimic, hsa-miR-7-5p inhibitor or hsa-miR-20b-5p inhibitor assessed by TUNEL staining. The data in the figure were measurement data and expressed as mean ± standard deviation. The data between multiple groups were analyzed by a one-way analysis of variance (Dunnett’s post hoc test). * *p* < 0.05. Cell experiments were repeated 3 times independently.
